# Prevalence, Risk Factors, and Molecular Epidemiology of Intestinal Carbapenem-Resistant Pseudomonas aeruginosa

**DOI:** 10.1128/Spectrum.01344-21

**Published:** 2021-11-24

**Authors:** Yanyan Hu, Yan Qing, Jiawei Chen, Congcong Liu, Jiayue Lu, Qi Wang, Shufang Zhen, Hongwei Zhou, Ling Huang, Rong Zhang

**Affiliations:** a Clinical Microbiology Laboratory, The Second Affiliated Hospital of Zhejiang University, School of Medicine, Zhejiang University, Hangzhou, China; b Department of Clinical Laboratory, Jinhua People’s Hospital, Jinhua, China; c Department of Clinical Laboratory Medicine, Maternal and Child Health Hospital of Linping District, Hangzhou, China; Keck School of Medicine of the University of Southern California

**Keywords:** prevalence, risk factors, clinical characteristics, *Pseudomonas aeruginosa*, CRPA, KPC

## Abstract

Pseudomonas aeruginosa may become multidrug-resistant (MDR) due to multiple inherited and acquired resistance mechanisms. The human gastrointestinal tract is known as a reservoir of P. aeruginosa and its resistance genes. In this study, we collected 76 intestinal carbapenem-resistant P. aeruginosa (CRPA) strains from clinical inpatients admitted to our hospital from 2014 to 2019, together with their medical data. We aim to analyze the clinical risk factors associated with CRPA infection and its molecular features. We found that the prevalence of CRPA in P. aeruginosa strains was 41.3% (95% confidence interval [CI], 34.1 to 48.8%). We also identified four variables associated with intestinal CRPA positivity, prior antibiotic exposure to aminoglycosides or carbapenems, underlying diabetes mellitus, and extraintestinal P. aeruginosa isolation. *bla*_KPC-2_ is the only detected carbapenemase gene, accounting for 21.1% of CRPA strains. The genetic environment showed that the *bla*_KPC-2_ gene was flanked immediately by IS*Kpn8* and IS*Kpn6* and several other mobile elements further upstream or downstream. Four sequence types (STs) were identified, with ST463 as the dominant sequence type. In conclusion, screening for P. aeruginosa colonization upon hospital admission could reduce the risk of P. aeruginosa infection and spread of CRPA in the hospital.

**IMPORTANCE** Pseudomonas aeruginosa may become multidrug-resistant (MDR) due to multiple inherited and acquired resistance mechanisms. The human gastrointestinal tract is known as a reservoir of P. aeruginosa and its resistance genes. Risk factor analysis and molecular epidemiology are critical for preventing their potential dissemination. Here, we identified four risk factors associated with intestinal CRPA—prior antibiotic exposure to aminoglycosides or carbapenems, underlying diabetes mellitus, and extraintestinal P. aeruginosa isolation. Further, we found similar genetic environments with several mobile elements surrounding the *bla*_KPC_ gene, a carbapenemase gene only detected in intestinal CRPA strains in this study. These findings are of significant public health importance, as the information will facilitate the control of the emergence and spread of CRPA.

## INTRODUCTION

Pseudomonas aeruginosa is a major opportunistic pathogen, recognized as one of the most common hospital-acquired pathogens worldwide (1). It is widely distributed in the environment due to its strong adaptability (2). The human intestine is a common reservoir of P. aeruginosa, which may cause infections of other patients through feces, contaminated medical equipment, and the environment (3) and which may be disseminated to other body sites and cause endogenous infection (4), especially in immunocompromised patients. Markou et al. found that P. aeruginosa can cause pulmonary infection through the oral-fecal route of transmission or spread from the intestine to the lungs through the blood and therefore cause pulmonary infection (5).

P. aeruginosa is resistant to most antibiotics due to its inherent and acquired resistance mechanisms. Carbapenems are the most effective antimicrobial agents against severe P. aeruginosa nosocomial infections involving bacteria producing cephalosporinase AmpC or extended-spectrum β-lactamases (6). However, carbapenem-resistant P. aeruginosa (CRPA) has already posed a significant threat to public health worldwide. The prevalence of CRPA has increased rapidly in recent years. The World Health Organization (WHO) ranked CRPA as one of the top critical pathogens in health care settings (7). A previous study reported that the intestinal prevalence of CRPA can reach 30% (8). However, systemic risk analysis and molecular epidemiology of CRPA collected from the gastrointestinal tracts of inpatients were rare in the domestic. In the current study, we identified risk factors associated with CRPA infection and the molecular epidemiology of intestinal CRPA strains to provide evidence-based recommendations for CRPA control.

## RESULTS

### Prevalence and risk factors of intestinal CRPA.

A total of 4,560 nonduplicated fecal samples were collected from January 2014 to December 2019, in which 184 strains were identified as P. aeruginosa. The 6-year overall isolation rate of P. aeruginosa from the gut is 4.0% (95% confidence interval [CI], 3.5 to 4.6%), and annually the prevalence of P. aeruginosa was 3.0% (95% CI, 1.7 to 4.3%) in 2014, 3.0% (95% CI, 1.9 to 4.0%) in 2015, 5.6% (95% CI, 3.9 to 7.3%) in 2016, 3.9% (95% CI, 2.7 to 5.1%) in 2017, 4.1% (95% CI, 2.8 to 5.4%) in 2018, and 6.2% (95% CI, 3.7 to 8.7%) in 2019 (Table 1). From January 2014 to December 2019, we isolated 184 P. aeruginosa strains from 184 nonduplicated fecal samples. Among these, 76 isolates were CRPA. The prevalence of CRPA in P. aeruginosa was 41.3% (95% CI, 34.1 to 48.8%). Ceftazidime/avibactam showed great activity against CRPA, followed by lipopeptides and aminoglycosides (Table 2). The MICs to carbapenems and other beta-lactams of 16 *bla*_KPC-2_-positive CRPA isolates were much higher than those of *bla*_KPC-2_-negative CRPA isolates (data not shown). However, both *bla*_KPC-2_-positive and -negative CRPA isolates were resistant.

**TABLE 1:** Annual prevalence of P. aeruginosa collected from feces of inpatients in Hangzhou, China

Yr	No. of samples	No. of P. aeruginosa isolates	No. of CRPA isolates	Isolation rate of P. aeruginosa (%) (95% CI)	Isolation rate of CRPA (%) (95% CI)
2014	670	20	9	3.0 (1.7–4.3)	1.3 (1.5–2.2)
2015	978	29	15	3.0 (1.9–4.0)	1.5 (0.8–2.3)
2016	699	39	17	5.6 (3.9–7.3)	2.4 (1.3–3.6)
2017	983	38	15	3.9 (2.7–5.1)	1.5 (0.8–2.3)
2018	876	36	11	4.1 (2.8–5.4)	1.3 (0.5–2.0)
2019	354	22	9	6.2 (3.7–8.7)	2.5 (0.9–4.2)
Total	4,560	184	76	4.0 (3.5–4.6)	1.7 (1.3–2.0)

**TABLE 2:** Antibiotic susceptibility profiles of 76 CRPA isolates^*a*^

Antibiotic	MIC_50_ (μg/mL)	MIC_90_ (μg/mL)	S (%) (no. of P. aeruginosa isolates)	I (%) (no. of P. aeruginosa isolates)	R (%) (no. of P. aeruginosa isolates)
IPM	8	256	2.6 (2)	10.5 (8)	86.8 (66)
MEM	8	256	14.5 (11)	23.7 (18)	61.8 (47)
CAZ	8	64	54.0 (41)	9.2 (7)	36.8 (28)
CAV	2	4	100.0 (76)	0.0 (0)	0.0 (0)
FEP	8	>256	52.6 (40)	11.8 (9)	35.5 (27)
TZP	16	256	51.3 (39)	13.2 (10)	35.5 (27)
ATM	16	>128	31.6 (24)	26.3 (20)	42.1 (32)
GM	1	32	88.2 (67)	0.0 (0)	11.8 (9)
AK	1	8	89.5 (68)	3.9 (3)	6.6 (5)
LEV	2	16	39.5 (30)	19.7 (15)	40.8 (31)
CIP	0.5	8	60.5 (46)	3.9 (3)	35.5 (27)
CO	1	1	97.4 (74)	0.0 (0)	2.6 (2)
PB	1	2	96.1 (73)	2.6 (2)	1.3 (1)

a IPM, imipenem; MEM, meropenem; CAZ, ceftazidime; CAV, ceftazidime-avibactam; FEP, cefepime; TZP, piperacillin-tazobactam; ATM, aztreonam; GM, gentamicin; AK, amikacin; LEV, levofloxacin; CIP, ciprofloxacin; CO, colistin; PB, polymyxin B; S, susceptible; I, intermediate; R, resistant.

A total of 61 potential risk factors in 10 categories were selected for risk analysis of intestinal CRPA. Among them, 23 significant factors showed a *P *value of <0.20 in the univariable analysis (Table 3) and were entered into the logistic regression model. In the multivariable analysis, four risk factors were identified to be associated with intestinal CRPA (*P < *0.05), including exposure to carbapenems (odds ratio [OR], 2.5; 95% CI, 1.1 to 5.5) or aminoglycosides (OR, 9.9; 95% CI, 1.9 to 51.4), underlying comorbidities with diabetes (OR, 3.9; 95% CI, 1.5 to 10.2), and P. aeruginosa carriage in nonfecal samples (OR, 3.4; 95% CI, 1.6 to 7.0) (Table 3). The Hosmer-Lemeshow test showed a *P* value of 0.981 (>0.05) with an area under the concentration-time curve (AUC) of 0.768 (>0.6; *P < *0.001).

**TABLE 3:** Analysis of risk factors associated with intestinal CRPA^*a*^

Variable^*c*^	Percentage (%)^*b*^	Prevalence of CRPA (%) (95% CI)	Univariate analysis		Multivariate analysis	
			OR (95% CI)	P*	OR (95% CI)	*P* ^#^
Age <60	35.8	33.3 (21.7–45.0)	1.0	0.101		
Age ≥60	64.2	45.8 (36.6–54.9)	1.7 (0.9–3.2)			
Type of antibiotic used in the past 3 mo (Yes/No)						
Penicillin and β-lactamase inhibitor combination	34.2	49.2 (36.5–61.9)	1.6 (0.9–3.0)	0.116		
Cephalosporin	50.5	35.5 (25.6–45.4)	0.6 (0.3–1.1)	0.114	0.5 (0.2–1.0)	0.051
Tigecycline	20.1	62.2 (45.8–78.6)	2.91 (1.4–6.1)	0.004		
Aminoglycosides	7.6	85.7 (64.7–100.0)	9.94 (2.2–45.8)	<0.001	9.9 (1.9–51.4)	0.006
Carbapenem	63.0	50.9 (41.6–60.1)	3.11 (1.6–6.0)	0.001	2.5 (1.1–5.5)	0.025
Vancomycin	34.2	50.8 (38.1–63.5)	1.8 (1.0–3.3)	0.059		
Antifungal agents	9.8	66.7 (42.5–90.8)	3.2 (1.1–8.9)	0.021		
Other potential risk factors (Yes/No)						
Extraintestinal PA	47.8	59.1 (48.6–69.6)	4.3 (2.3–8.1)	<0.001	3.4 (1.6–7.0)	0.001
Diabetes	16.3	56.7 (37.8–75.5)	2.1 (1.0–4.7)	0.062	3.9 (1.5–10.2)	0.006
Admitted to ICU in the past 3 mo (Yes/No)	20.1	51.4 (34.5–68.2)	1.7 (0.8–3.4)	0.165		
Samples collected from ICU	49.5	52.7 (42.3–63.2)	0.4 (0.2–0.7)	0.002		
Invasive procedures	78.3	45.8 (37.6–54.1)	2.5 (1.2–5.6)	0.018		
Malignant tumor	21.2	30.8 (15.6–45.9)	0.6 (0.3–1.2)	0.132		
CRKP infection history	21.2	51.3 (34.9–67.7)	1.1 (1.0–1.1)	0.007		
Retention catheter in the past 3 mo (Yes/No)						
Vascular catheter	76.1	47.1 (38.8–55.5)	3.0 (1.4–6.6)	0.004		
Urinary catheter	81.0	45.6 (37.5–53.7)	2.8 (1.2–6.6)	0.014		
Abdominal or pelvic catheter	34.2	31.7 (19.9–43.6)	0.5 (0.3–1.0)	0.057		
Artificial lung ventilation	72.3	47.4 (38.8–56.0)	2.6 (1.3–5.4)	0.007		
Other catheters	65.2	49.2 (40.1–58.2)	2.7 (1.4–5.2)	0.003		
Main diagnosis						
Digestive disease	35.3	23.1 (12.6–33.6)	0.3 (0.1–0.6)	<0.001		
Neurological disease	32.1	54.2 (41.1–67.3)	2.2 (1.2–4.1)	0.014		
Other indistinct disease	14.7	55.6 (35.5–75.6)	2.0 (0.9–4.5)	0.103		

a CI, confidence interval; OR, odds ratio; *, Significant variables in univariable analysis (*P* < 0.20) were entered into a logistic regression model; ^#^, Significant variables in multivariable analysis (*P* < 0.05) were identified as the risk factors in this study.

b Percentage refers to corresponding variable.

c “Yes/No” indicates the existence/absence of the corresponding variable.

### Molecular epidemiology of CRPA.

**FIG 1:**
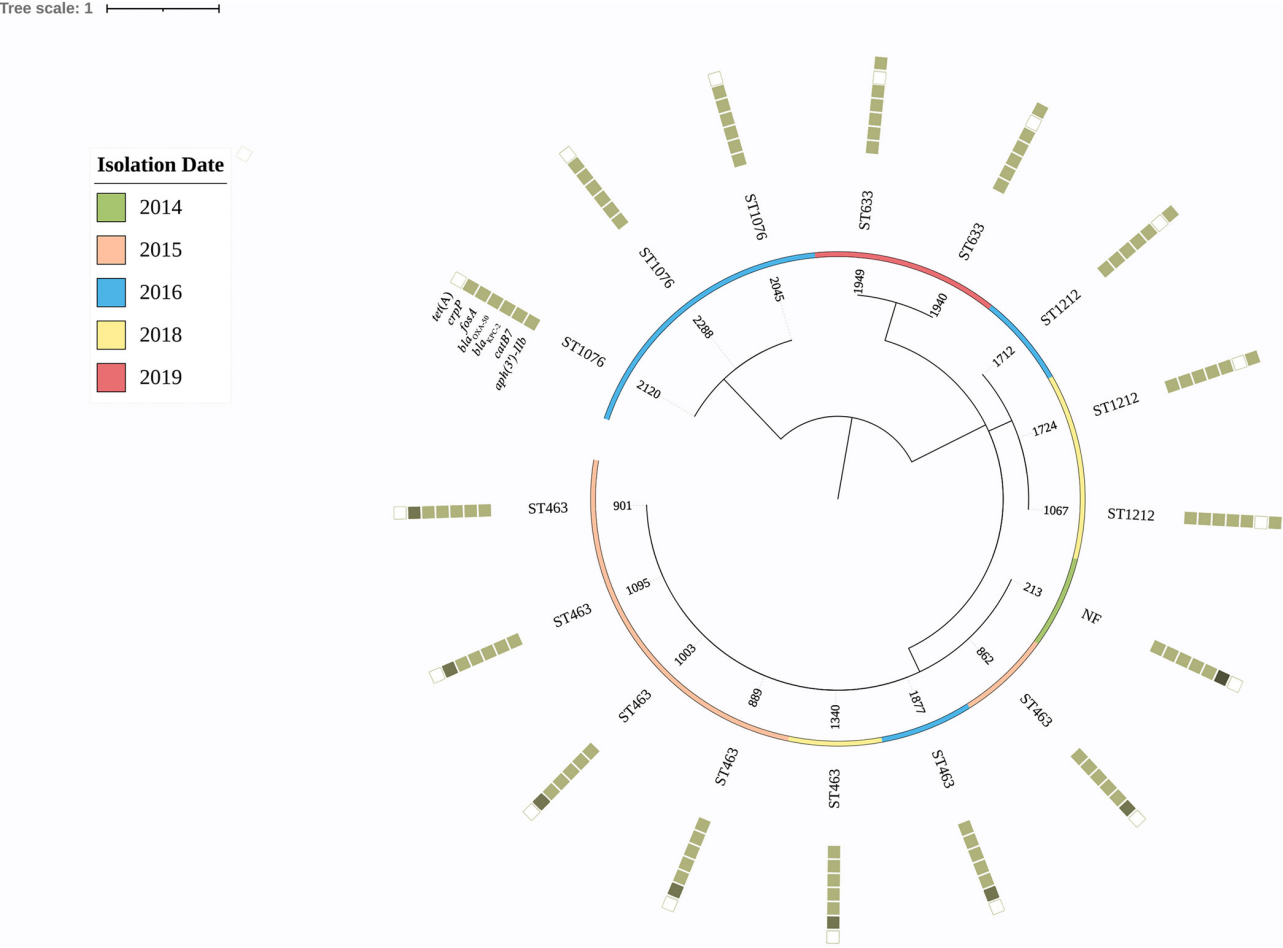
Phylogenetic tree of 16 KPC-producing P. aeruginosa strains, with their sequence types and profiles of inherent and acquired antimicrobial resistance genes. The isolation date of the isolates is illustrated. The rings consisting of small squares from the inside out indicated as *aph(3′)-IIb*, *catB7*, *bla*_KPC-2_, *bla*_OXA-50_, *fos*A, *crpP*, and *tetA*. The filled squares represent the presence of a resistance gene. The *crpP* gene ring, light- medium-, and dark-colored squares, represents one, two, and three copies of this gene, respectively.

A total of 16 P. aeruginosa isolates were positive for the simplified carbapenem inactivation method (sCIM), and PCR confirmed that these 16 strains all harbored the *bla*_KPC-2_ gene, accounting for 21.1% (16/76), and none of the other carbapenemase genes were detected. These 16 strains were distributed in the years 2014 (1 isolate), 2015 (5 isolates), 2016 (5 isolates), 2018 (3 isolates), and 2019 (2 isolates). Multilocus sequence type (MLST) analysis revealed four sequence types (STs) and an unknown ST in the 16 *bla*_KPC-2_ CRPA isolates (Fig. 1). ST463 was the dominant sequence type, accounting for 43.8% (7/16). Meanwhile, there is only one base discrepancy between ST463 and the unknown ST in the 440 bp of the housekeeping gene *mutL* (T to C).

Besides the *bla*_KPC-2_ gene, genome sequence analysis also identified seven other resistance genes in the 16 isolates (Fig. 1). Among them, five genes, namely, *aphs-IIb*, *catB7*, *fos*A, *bla*_PAO_, and *bla*_OXA-50_, are naturally chromosomally encoded in P. aeruginosa and were detected in all the isolates. The other two acquired genes were *crpP* (fluoroquinolone resistance gene) and *tetA* (tetracycline resistance gene), present in 68.8% (11 out of 16) and 31.3% (5 out of 16) of isolates, respectively. In addition, some strains harbored two to three copies of the *crpP* gene. Interestingly, none of the isolates harbored both *crpP* and *tetA* genes.

**FIG 2:**
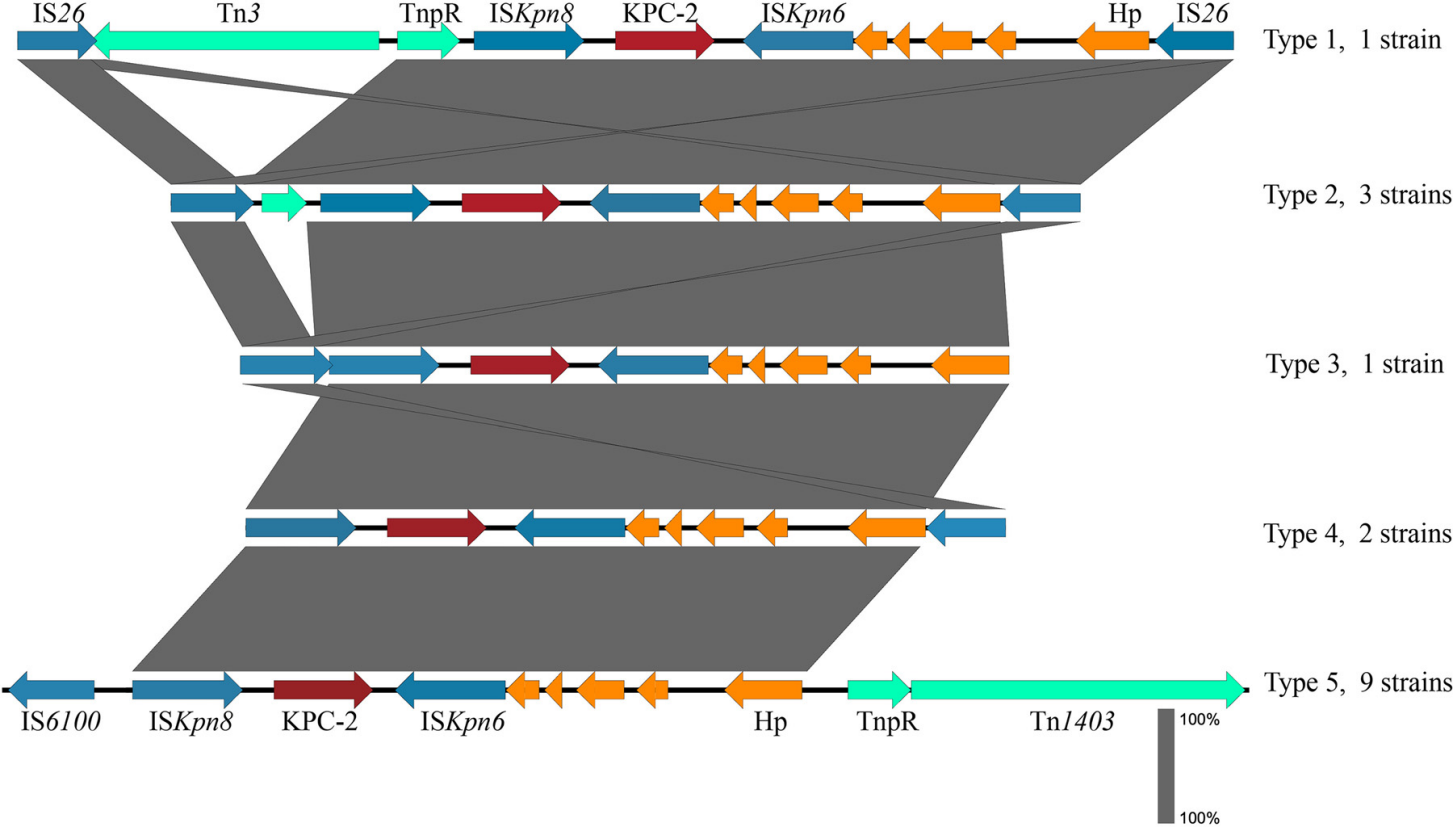
The diverse genetic context of gene *bla*_KPC-2_. Transposons, ISs, *bla*_KPC-2_, and hypothetical protein are colored in green, blue, red, and orange, respectively. Five types were identified, and the IS*Kpn8*-*bla*_KPC-2_-IS*Kpn6* unit was conserved in all the isolates.

Genetic environment analysis showed that *bla*_KPC-2_ was flanked immediately by IS*Kpn8* (upstream) and IS*Kpn6* (downstream) and several mobile elements, including IS*26*, IS*6100*, Tn*3*, and TnpR further upstream and IS*26*, Tn*1403*, and TnpR further downstream (Fig. 2). The patterns of mobile elements in the *bla*_KPC-2_ genetic context were classified into five types, among which, four belonged to the seven ST463 strains; for the other nine non-ST463 strains, they all showed a conserved IS*6100*-IS*Kpn8*-*bla*_KPC-2_-IS*Kpn6-*TnpR-Tn*1403* genetic context (Fig. 2).

## DISCUSSION

The intestine is a major reservoir of P. aeruginosa and its resistance genes. A prospective survey of P. aeruginosa colonization and infection in the intensive care unit showed that P. aeruginosa was mainly endogenous. Additionally, P. aeruginosa strains were generally patient- and site-specific, especially in the gastrointestinal tract (9). The prevalence of CRPA among intestinal P. aeruginosa isolates in our hospital was 41.3% (95% CI, 34.1 to 48.8%), similar to those from extraintestinal CRPA reported in our previous study (36.7%; 95% CI, 37.3 to 37.8%) (10) but higher than the 25.3% (95% CI, 18.3 to 32.4%) intestinal CRPA prevalence reported in another study (11).

Understanding the risk factors for the occurrence of CRPA is critical for infection prevention and management. Previous reports on the risk factors have shown that prior antibiotic exposure, especially carbapenems, is often regarded as a risk factor for the intestinal carriage of CRPA (8, 11). Here, prior usage of not only carbapenems but also aminoglycosides was identified as a risk factor for intestinal CRPA infections. Similarly, previous studies (12, 13) also identified aminoglycosides are associated with the occurrence of CRPA. However, there is a lack of specific evidence for aminoglycoside-induced CRPA. Hence, this result will need to be validated by other studies. Nevertheless, the result suggests that clinicians should pay attention to empirical antibiotic treatment with carbapenems and aminoglycosides.

Underlying diabetes mellitus and extraintestinal P. aeruginosa isolation are two other risk factors found in our study, which were barely reported in previous studies. Rattanaumpawan et al. (14) reported that diabetes mellitus is associated with the incidence of extended-spectrum β-lactamase (ESBL)-producing *Enterobacteriaceae*, indicating the finding that underlying diabetes mellitus might be a potential predictor of intestinal CRPA infection. It is highly recommended to continue the study to clarify the risks of underlying diabetes mellitus and extraintestinal P. aeruginosa isolation to intestinal CRPA. A systematic review reported that intensive care unit (ICU) stay was one of the most significant risk factors for the acquisition of CRPA (15). However, we did not identify such an association, possibly because we focused on the intestinal CRPA instead of other body sites. This result is consistent with the intestinal CRKP risk factor investigation in our hospital (data not published).

In terms of mechanisms of carbapenem resistance, the metallo-β-lactamases are the most commonly observed carbapenemases, with Verona integron-encoded metallo-β-lactamase (VIM) and imipenemase (IMP) types being the most widely distributed geographically (16). However, *bla*_KPC-2_ is the main carbapenemase gene in both fecal samples in the current study and other specimens in our hospital (17). KPC-type carbapenemases have been predominantly found in K. pneumoniae, but less frequently in P. aeruginosa, especially gastrointestinal ones. Our previous report (17) on clinical samples showed that the *bla*_KPC-2_ gene is usually located in plasmids with mobile elements (i.e., insertion sequences [IS] and transposons), as is the *bla*_KPC-2_ gene detected from the fecal samples in the current study, making it potentially transferable in the hospital environment or *in vivo* translocation. In the current study, ceftazidime/avibactam showed excellent activity (100% susceptible) against CRPA, a little higher than previous reports, which might due to the mechanisms of carbapenem resistance. None of the metallo-β-lactamase (naturally resistant to ceftazidime/avibactam) was detected in the 76 intestinal CRPA strains. In addition, we found 2.6% imipenem-susceptible and 14.5% meropenem-susceptible CRPA strains. These imipenem-resistant but meropenem-susceptible (IRMS) and meropenem-resistant but imipenem-susceptible (MRIS) phenotypes may be due to the mutations of the *oprD* gene in IRMS strains and overexpression of mexAB efflux pumps in MRIS strains, respectively (18), and these two mechanisms may also cause carbapenem-resistant but cephalosporin-susceptible phenotypes among non-carbapenemase-producing CRPA (19). The susceptible rates of cephalosporins and piperacillin-tazobactam were similar to those in our previous survey of clinical imipenem-resistant P. aeruginosa strains (10). These findings confirm the strong contribution of mutation-driven mechanisms among non-carbapenemase-producing CRPA strains.

ST463, a potential high-risk clone of P. aeruginosa (20), is the dominant CRPA clone in our hospital (17). Here, we also found ST463 CRPA as the dominant clone (43.8%, 7/16) in fecal samples, indicating that the high-risk clone ST463 might colonize in the intestinal tract, which may cause infections in patients with a suppressed immune system. Thus, prevention of CRPA colonization-acquisition represents an important target for interventions to reduce infection and spread of CRPA in the hospital.

Our study had several limitations. (i) The sample size of this study is limited to 184 hospitalized patients in a single hospital, which can be biased. (ii) We did not have data regarding medications or swabs before hospital admission. Fecal samples were collected during the hospital stay but not on hospital admission. (iii) Only production of carbapenemases was analyzed in the molecular mechanisms of carbapenem resistance among the CRPA isolates, while chromosomal mutations (such as those leading to the loss or inactivation of the OprD porin and/or the overexpression of efflux pumps) were not included.

In summary, this is the first systematic risk factor analysis of intestinal CRPA in China. Our results revealed that prior antibiotic exposure of aminoglycosides or carbapenems, underlying diabetes mellitus, and extraintestinal P. aeruginosa isolation are associated with the prevalence of intestinal CRPA. KPC is the main carbapenemase detected in the intestinal CRPA. Reducing intestinal flora exposure to antibiotics is a major issue in the control of the emergence and spread of CRPA. Empirical carbapenem or aminoglycoside treatments should be initiated only when necessary.

## MATERIALS AND METHODS

### Strains and antimicrobial susceptibility testing.

Samples were collected in the Second Affiliated Hospital of Zhejiang University School of Medicine (SAHZU), a general hospital with 3,200 beds. Fecal samples were cultured on blood agar plates and Salmonella *Shigella* (SS) agar, followed by incubation overnight at 35°C. The following day, colonies on blood and SS agar were all identified by matrix-assisted laser desorption ionization–time-of-flight mass spectrometry (MALDI-TOFMS) (Microflex LT; Bruker Daltonik GmbH, Bremen, Germany), and fecal samples containing P. aeruginosa strains were defined as intestinal P. aeruginosa carriage cases. From January 2014 to December 2019, a total of 184 nonduplicated P. aeruginosa isolates were collected from the fecal samples of inpatients. The average age of these 184 inpatients was 62.7 years old, and 67.9% of them were male. The average number of hospitalization days of inpatients before P. aeruginosa was isolated was 21.1 days; 91 (49.4%) patients were in ICUs, and 119 (64.7%) were from urban areas (see Table S1 in the supplemental material). All the strains were screened using the imipenem and meropenem disk diffusion method. Isolates were identified to be CRPA if they were resistant to imipenem or meropenem. These strains were then tested for susceptibility to carbapenems (imipenem and meropenem), β-lactam combination agents (piperacillin/tazobactam, ceftazidime/avibactam), cephems (ceftazidime and cefepime), monobactams (aztreonam), lipopeptides (colistin and polymyxin B), aminoglycosides (gentamicin and amikacin), and fluoroquinolones (ciprofloxacin and levofloxacin) using the broth microdilution method and interpreted according to the CLSI M100-S29 guideline for P. aeruginosa (21).

### Detection of carbapenemases.

For CRPA strains, the sCIM was used to screen the carbapenemase-positive strains according to the reference (22). Meanwhile, PCR was used to amplify common carbapenemase-resistant genes as described previously, including *bla*_KPC_ (23), *bla*_NDM-1_ (24), *bla*_IMP_, and *bla*_VIM_ (25).

### Genome sequencing.

The genomic DNA of carbapenemase-positive isolates was extracted using a PureLink Genomic DNA minikit (Invitrogen, Carlsbad, CA, USA) according to the manufacturer’s instructions. Indexed sequencing libraries were prepared using a TruSeq DNA PCR-free sample preparation kit (Illumina, Inc., San Diego, CA) according to standard protocols. Libraries were sequenced on the Illumina HiSeq X10 platform with a 150-bp paired-end strategy. Raw reads were trimmed and assembled to contigs using SPAdes version 3.11.1 (26). Acquired carbapenem resistance genes and multilocus sequence types (MLST) were determined via the Center for Genomic Epidemiology website to screen for the presence of acquired antimicrobial resistance genes (ARGs) (27) and the sequence type (28), respectively. A core-genome-based phylogenetic tree was generated using Parsnp in the Harvest package (29). The molecular features of each isolate were visualized using the online tool iTOL (30). To analyze the genetic environment of the *bla*_KPC-2_ gene, *bla*_KPC-2_-containing contigs were further annotated using RAST (31) and examined manually. For strains with missing sequences around *bla*_KPC-2_, a PCR mapping approach was adopted to compare the genetic context of the *bla*_KPC_ gene with that found in plasmids pPA1011 (MH734334) and p14057 (KY296095), which are the most prevalent genetics in our previous article (17). A series of primers were designed at the base of *bla*_KPC_-surrounding sequences (Table S2, Fig. S1). PCR experiments were performed according to standard conditions. The obtained amplification products were then sequenced.

### Statistical analysis.

Clinical information of inpatients with intestinal carriage of P. aeruginosa was collected via the hospital information system. The Pearson chi-square (χ^2^) test and Fisher’s exact test were used to calculate the statistical significance. Risk analyses were performed using SPSS version 23. In the univariable analysis, factors with a *P *value of <0.20 were considered significant and then selected into a logistic regression model using the likelihood ratio method. Factors with a *P *value of <0.05 were considered the risk factors in this study. The Hosmer-Lemeshow test was used for goodness of fit for the logistic regression model.

### Data availability.

The Illumina sequences generated and used in this study are deposited and available at the NCBI under BioProject number PRJNA767942. All 16 P. aeruginosa isolates are available under BioSample accession numbers SAMN21988683 to SAMN21988698. All other data generated or analyzed during this study are included in this article and its supplementary files.
