# Supplementary material for: Prevalence, Risk Factors, and Molecular Epidemiology of Intestinal Carbapenem-Resistant Pseudomonas aeruginosa
Source: Microbiol Spectr. 2021 Nov 24;9(3):e01344-21. doi: 10.1128/Spectrum.01344-21 (PMC8612150; doi:10.1128/Spectrum.01344-21)
Supplement: SUPPLEMENTAL FILE 2 — Supplemental material. Download SPECTRUM01344-21_Supp_2_seq4.pdf, PDF file, 0.2 MB [file spectrum01344-21_supp_2_seq4.pdf]

Table S2. Primers for PCR amplification of the *bla*$_{KPC}$ surrounding sequences

Primer name	No. in Fig. S1	Primer sequence (5'-3')
1011-IS*26*	1F	AACGCGGAGTGAATGTCGAT
1011-Tn*3* transposase	1R	CTGCTTACCAGGCGCATTTC
1011-Tn*3* transposase	2F	TCCAGAAGGCCTTCCAGCACA
1011-Tn*3* resolvase	2R	AACCGAACCGCGACACCCTGA
1011- Tn*3* resolvase	3F	ACAAGGCATCCGGCAGTTCAA
1011-IS*Kpn8* transposase	3R	CGCAGCGTCGATTCCAGTTC
1011-IS*Kpn8* transposase	4F	GTGGAGCTGCGCAAGACGTT
1011-KPC-2	4R	CCAGACGACGGCATAGTCAT
1011-KPC-2	5F	CGGAGACAAAACCGGAACCT
1011-IS*Kpn6*	5R	GACCACAAGGCGATGAGCTA
1011-hp	6F	AGGCGATACCATTTAGCCCG
1011-IS*26*	6R	AACGCGGAGTGAATGTCGAT
14057-IS*Kpn8*	7F	AGCTTGACGATCTTGACGGG
14057-IS*6100*	7R	TCGAATCCCTTGATCGTGGC
14057-Tn*1403* transposase	8F	GTTGTAGGTAGGTGCGCAGT
14057-hp	8R	AGGCGATACCATTTAGCCCG
novel-IS*26*	9F	ACGATTTACCGCTGGGTTCA
novel-IS*Kpn8*	9R	GCTTGACGATCTTGACGGGA

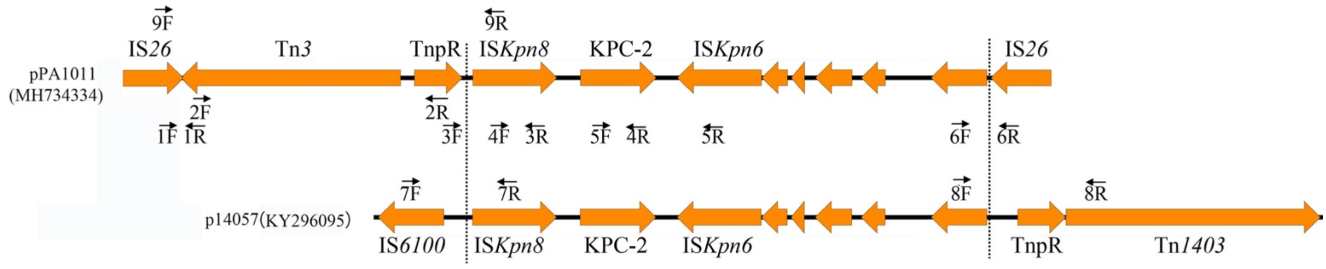

Fig. S1: Genetic environment of *bla*$_{KPC-2}$ in pPA1011 and p14057.
